# Alphaviruses in Gene Therapy

**DOI:** 10.3390/v7052321

**Published:** 2015-05-07

**Authors:** Kenneth Lundstrom

**Affiliations:** PanTherapeutics, Lutry CH1095, Switzerland; E-Mail: lundstromkenneth@gmail.com; Tel.: +41-79-776-6351

**Keywords:** alpahvirus, vector delivery, cancer, vaccines, gene therapy, RNA interference, micro-RNA

## Abstract

Alphavirus vectors present an attractive approach for gene therapy applications due to the rapid and simple recombinant virus particle production and their broad range of mammalian host cell transduction. Mainly three types of alphavirus vectors, namely naked RNA, recombinant particles and DNA/RNA layered vectors, have been subjected to preclinical studies with the goal of achieving prophylactic or therapeutic efficacy, particularly in oncology. In this context, immunization with alphavirus vectors has provided protection against challenges with tumor cells. Moreover, alphavirus intratumoral and systemic delivery has demonstrated substantial tumor regression and significant prolonged survival rates in various animal tumor models. Recent discoveries of the strong association of RNA interference and disease have accelerated gene therapy based approaches, where alphavirus-based gene delivery can play an important role.

## 1. Introduction

**Figure 1:**
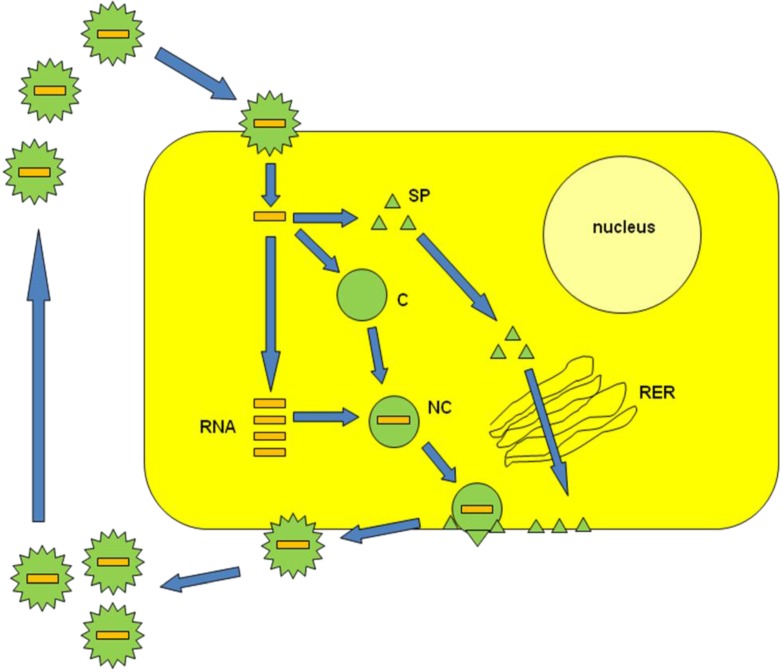
Alphavirus life-cycle. Alphavirus particles enter host cells through fusion with the plasma membrane and RNA is released into the cytoplasm. Capsid (C) and spike proteins (SP) are expressed. New copies of RNA form nucleocapids (NC) with C and are transported to the plasma membrane. Simultaneously, the SPs travel through the rough endoplasmic reticulum (RER) before being assembled with NCs. Mature viral particles are released through budding.

Alphaviruses are single-stranded RNA viruses belonging to the *Togaviridae* family [1]. The enveloped alphavirus particles are composed of a protein capsid structure surrounded by spike membrane proteins. Alphaviruses recognize surface proteins such as laminin and heparin receptors on mammalian and insect cells, which results in delivery of the RNA genome to the cell cytoplasm for immediate RNA replication (Figure 1) [2]. New progeny RNA is packaged into nucleocapsids, which are transported to the plasma membrane and coated by membrane proteins for release of viral particles by budding. A number of alphaviruses have been the cause of fever epidemics in Africa [3,4]. For this reason, engineering of alphavirus expression vectors is generally based on attenuated or avirulent strains to provide the highest possible biosafety level. Alphavirus vectors have been regularly applied for the expression of heterologous recombinant proteins in mammalian cell lines [5], primary cells [6] and *in vivo* [7]. A variety of studies conducted in animal tumor models has demonstrated substantial tumor regression [8] and prophylactic immunization with alphavirus vectors expressing tumor antigens has showed protection against tumor challenges [9]. Moreover, the discovery of a clear link between RNA interference (RNAi) and disease has increased interest in using viral vectors for gene therapy applications [10]. In this review, the various types of alphavirus vectors are described including their applications in gene therapy.

## 2. Alphavirus Vector Systems

**Figure 2:**
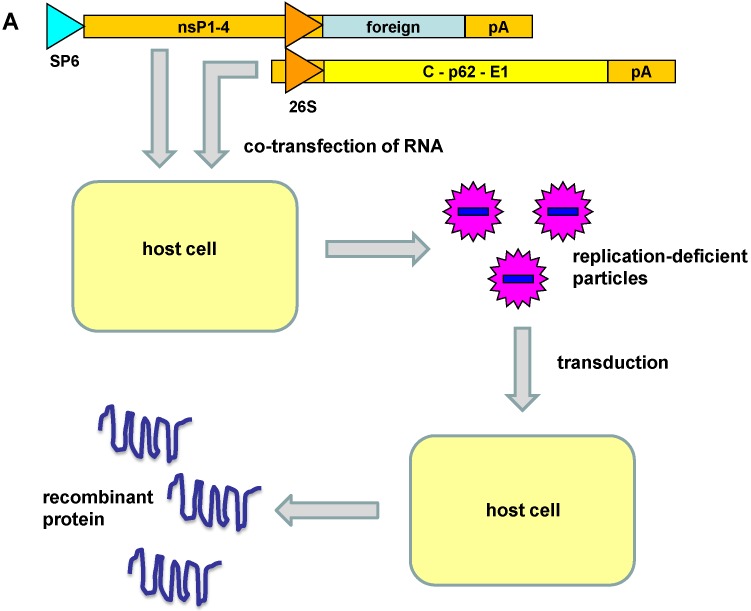

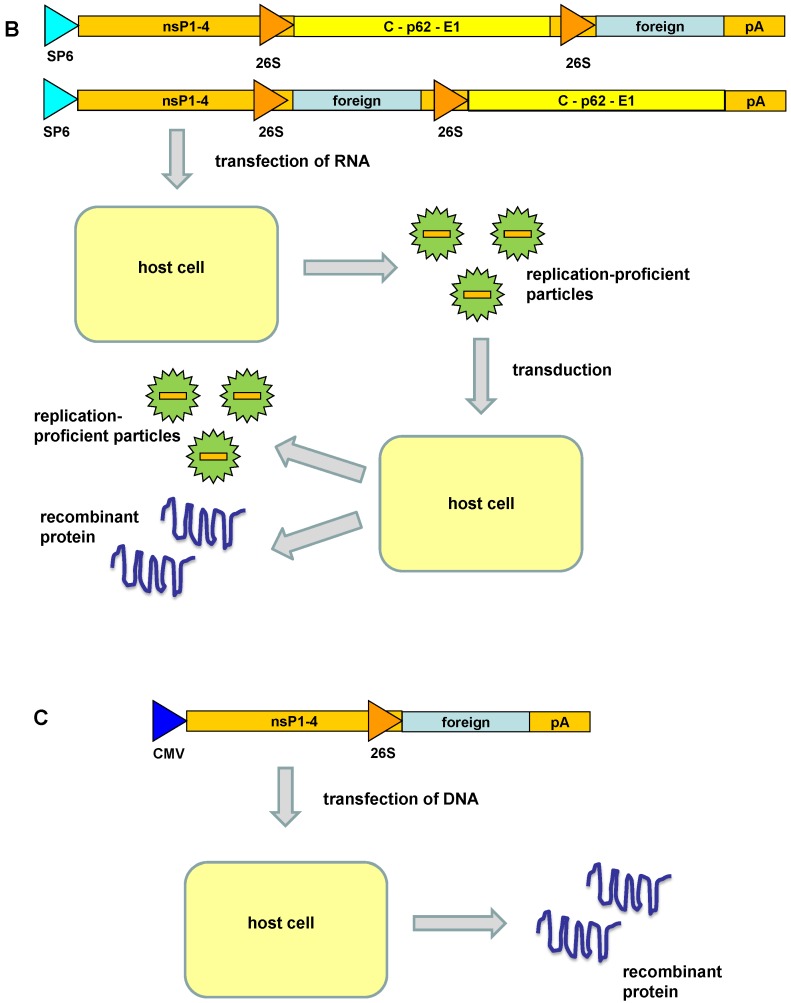
Alphavirus vector systems. (**A**) Replication-deficient recombinant virus particles: recombinant particles are generated by co-transfection of *in vitro* transcribed RNA from expression and helper vectors. The replication-deficient particles are capable of transduction of host cells resulting in high-level transient expression of recombinant protein. (**B**) Replication-proficient recombinant virus particles: transfection of RNA including the full-length alphavirus genome and the gene of interest generates replication-proficient particles capable of recombinant protein expression and simultaneous virus production after transduction of host cells. (**C**) Layered DNA/RNA vectors: transfection of vector DNA provides transient expression of recombinant protein.

Due to the potential pathogenicity of alphaviruses in general, vectors for gene delivery and expression have been based on avirulent or attenuated strains. Mainly, Semliki Forest virus (SFV) [5], Sindbis virus (SIN) [11] and Venezuelan equine encephalitis virus (VEE) [12] have been subjected to vector engineering. In this context, gene delivery systems have been developed for naked RNA, plasmid DNA and recombinant viral particles, which has culminated in the engineering of different types of vectors (Figure 2). 1. Replication-deficient recombinant virus particles: the gene of interest is inserted in the alphavirus expression vector downstream of the subgenomic 26S promoter. Expression of the alphavirus nonstructural genes generates the replicase complex responsible for highly efficient RNA replication. Production of recombinant viral particles is possible by providing the alphavirus structural proteins in *trans* from a helper vector. High-titer replication-deficient particles can be generated from *in vitro* transcribed RNA from expression and helper vectors co-transfected into mammalian host cells. Direct transfection of layered DNA/RNA expression and helper plasmid vectors (see below) are also able to generate recombinant particles, however, with 10–100 fold lower titers [13]. 2. Replication-proficient recombinant virus particles: vectors are based on the full-length alphavirus genome in which a second subgenomic promoter has been inserted either upstream or downstream of the alphavirus structural genes [14]. *In vitro* transcribed RNA is transfected into mammalian host cells for the generation of recombinant replication-proficient particles. 3. Layered DNA/RNA vectors: by replacing the SP6 RNA polymerase promoter with a CMV promoter, plasmid DNA can be directly transfected into mammalian cells for recombinant protein expression. Co-transfection with CMV promoter-engineered helper vector allows production of replication-deficient viral particles. The alphavirus vector systems described above permit different approaches, depending on the selected application. In this context, local expression is achieved *in vivo* by administration of replication-deficient vectors. In case extended spread of expression is requested it is favorable to use replication-proficient virus particles. As far as vaccine development, plasmid DNA vectors have been commonly used [15]. The choice of alphavirus vectors is further enhanced by the possibility to use *in vitro* transcribed RNA for immunization [16].

## 3. Engineered Alphavirus Vectors

In attempts to enhance the heterologous gene expression levels, reduce the cell cytotoxicity and improve biosafety, a number of modified alphavirus vectors have been engineered. For SFV, the introduction of three point mutations in the p62 precursor sequence resulted in the second-generation helper vector pSFV-helper2, which rendered conditionally infectious particles [17]. Infectious particles can be obtained only after activation with α-chymotrypsin, which substantially reduces the frequency of recombination as a means of generating replication-proficient infectious particles. Furthermore, split helper vectors where the capsid and envelope genes are provided from separate helper vectors have been engineered for SFV [18] and SIN [19]. This approach significantly reduces recombination events.

A number of modifications have also been made to both SFV and SIN expression vectors. In attempts to enhance transgene expression, the translation enhancement signal of the capsid protein was introduced into the SFV expression vector, which resulted in 5–10-fold increase in protein expression levels [20]. Furthermore, introduction of the FMDV 2A protease sequence provided appropriate cleavage of the enhancer sequence from the final gene product. In attempts to reduce the cytopathic effect of alphavirus vectors, mutations have been introduced into the nonstructural genes. Both engineered SFV [21,22] and SIN [23,24] mutant vectors have been demonstrated to cause lower toxicity to host cells and have managed to provide enhanced expression levels for a prolonged time. Moreover, a single mutation in the SIN nsP2 gene resulted in persistent infection of transduced host cells [25]. In another approach, engineering of an SFV expression vector based on the avirulent strain A7(74) resulted in significantly reduced host cell cytotoxicity and extended transgene expression [26,27]. Recently, enhanced protein expression was achieved by designing VEE self-replicating subgenomic RNA replicons [28]. This approach allows the additional amplification of underused VEE replicon enzymes and resulted in 10–50-fold increase in protein expression.

Other vector designs relate to delivery and encompass generation of virus-like particles (VLPs) and encapsulation of viral particles. In this context, SIN and Ross River virus (RRV) nucleocapsids have been produced in a T7-based *E. coli* expression system [29]. Importantly, oligomerization of capsid protein occurred only in the presence of single-stranded RNA and not double-stranded nucleic acid. The encapsulation technology has allowed packaging of short single-stranded DNA and RNA molecules, small fluorescent-labeled oligonucleotides, and gold particles [30,31]. Moreover, liposome-encapsulated SFV particles carrying the LacZ gene demonstrated enhanced β-galactosidase expression in LNCaP tumors in a prostate cancer mouse model [8]. Encapsulated SFV-IL-12 particles also showed 5–10-fold increase in IL-12 serum levels after systemic administration to kidney carcinoma and melanoma patients [32].

## 4. Alphavirus Vectors in Gene Therapy

The broad host range and high level of transient heterologous gene expression are features, which have made alphaviruses attractive for gene therapy applications. Particularly, cancer therapy has been the main area of interest.

### 4.1. Tumor Targeting and Cancer Therapy

Although the broad host range can be advantageous, it also raises concerns in case of cancer therapy where systemic delivery targets not only cancer cells but also normal tissue. In attempts to improve tumor targeting, IgG binding domains of protein A were engineered into the SIN E2 envelope protein, which enhanced substantially transduction of host cells treated with a monoclonal antibody against surface proteins [33]. At the same time, a 10^5^-fold reduction in BHK cell transduction was observed. More recently, natural tumor targeting of SIN particles was discovered after systemic delivery in mice implanted with human xenografts [34]. This was further confirmed by intraperitoneal administration of SIN particles expressing luciferase, which resulted in targeted reporter gene expression in fibrosarcomas in the tail of the injected mice. Interestingly, similar experiments with SFV particles did not show any tumor targeting [35]. A closer evaluation of SIN particle tumor tropism in human xenograft models suggested that SIN transduction was not defined by SIN receptor levels, but by the interferon (IFN) response in tumors since cells with defects in either IFN production or signaling show strong susceptibility to SIN particles [36].

Another approach to achieve tumor targeting has been the engineering of liposome encapsulated nucleic acids and viral particles. As briefly described in the section on alphavirus vectors, systemic delivery of liposome-encapsulated SFV particles showed enhanced reporter gene expression in tumor tissue due to passive targeting [8] and allowed conducting a phase I clinical trial on kidney carcinoma and melanoma patients using interleukin-12 (IL-12) as the therapeutic gene [32]. The outcome was 5–10-fold increase in plasma levels of IL-12; a maximum tolerated dose (MTD) of 3 × 10^9^ encapsulated SFV particles; no kidney, liver, bone marrow, neuro- or cardio-toxicity; and the possibility of conducting repeated particle administration without any substantial immune response against liposomes or SFV. Furthermore, SIN replicon RNA-liposome nanoparticles have provided enhanced reporter gene expression and immune responses compared to naked RNA [37], which makes this approach attractive for vaccine development.

In the context of cancer therapy, alphavirus vectors have been applied for direct intratumoral administration, initially with reporter genes, followed by application of therapeutic genes. Administration of SFV particles expressing the p40 and p35 subunits of IL-12 showed substantial tumor regression and inhibition of tumor blood vessel formation in a mouse B16 tumor model [38]. Likewise, when severe combined immunedeficieny (SCID) mice with human lung tumor xenografts were subjected to intratumoral injection with SFV-GFP particles, significant tumor regression was observed [39]. Application of SFV-IL-12 vectors with the translation enhancement signal from the capsid gene showed complete regression of K-BALB tumors in BALB/c mice [40]. Likewise, therapeutic efficacy was achieved in mice implanted with CT26 tumors after injection of SFV-IL-18 vectors [41]. Moreover, when the vascular endothelial growth factor receptor-2 (VEGFR-2) was expressed from SFV vectors reduced growth of tumors and metastases was observed in mice [42].

In attempts to improve vector distribution, replication-proficient oncolytic vectors based on the avirulent SFV strain A7(74) have been engineered leading to lysis of several cancer cell lines [43]. A single injection of SCID mice implanted with human melanoma xenografts with 10^6^ particles resulted in strong tumor regression. In another study, oncolytic SFV A7(74)-EGFP vectors also demonstrated significant tumor shrinkage in a mouse model for human osteosarcoma [44]. Moreover, in animals with highly aggressive tumors, extended survival was observed. Also, in comparison to the conditionally replicating adenovirus Ad5-∆24TK-GFP the survival rate was significantly increased after local administration of oncolytic SFV-EGFP in mice with orthotypic lung cancer xenografts [45].

### 4.2. Cancer Vaccines

Alphavirus vectors have been subjected to cancer vaccine development as naked RNA, recombinant viral particles and plasmid DNA [46]. For example, immunization of mice with SFV particles expressing the P1A gene coding for the weak transplantation antigen P815A, protected against P815A tumor challenges [47]. Other examples of particle vaccinations are VEE delivery of the HPV-E7 gene [48] and immunization with SFV particles expressing B16 and 203 tumor antigens [49]. In both cases protection against tumor challenges was achieved in mouse models. VEE particles have shown high susceptibility of dendritic cells (DCs) and transduction of murine DCs resulted in high-level expression of the truncated neu oncogene, which induced robust neu-specific CD8^+^ T-cell and anti-neu IgG responses [50]. A single immunization resulted in the regression of large established tumors in mice [50].

In addition to recombinant particles, layered DNA vectors have been applied for immunization studies. For example, when mice immunized with SIN DNA vectors expressing the murine melanoma cell adhesion molecule (MCAM/MUC18), protection against lethal challenges was obtained [51]. Similarly, when mice were vaccinated intramuscularly with the DNA-based SIN vector expressing the neu gene 14 days prior to injection of tumor cells, strong protection against tumor development was observed [52]. In another study, immunization with SIN-HER2/neu DNA vectors provided protection against tumor challenges in a mouse breast tumor model [53]. Furthermore, intramuscular injection of SIN DNA encoding gp100 and IL-18 induced antigen-specific immune responses against malignant brain tumors [54]. Interestingly, comparison of SIN DNA replicon vectors to conventional DNA plasmids indicated that similar immune responses were elicited by doses 100–1000-fold lower for the SIN DNA replicon after intramuscular administration [55].

An interesting approach has been to apply *in vitro* transcribed RNA replicons for vaccination. In this context, when 1 μg of SFV RNA replicons expressing bacterial β-galactosidase was used for immunization of mice, therapeutic efficacy and complete tumor protection was obtained [16]. The survival rate was extended in vaccinated mice by 10–20 days in comparison to control animals. Moreover, RNA replicons have been applied for vaccine studies with secreted alkaline phosphatase (SEAP) as a reporter gene [37]. However, although immune responses were obtained, RNA-lipid nanoparticles generated superior immune responses in comparison to naked RNA.

### 4.3. RNA Interference Approaches

The influence of gene expression regulation on gene therapy has become even more important with the discovery of the involvement of RNAi and particularly micro RNA (miRNA) [10]. RNAi was demonstrated to efficiently inhibit SFV replication by expression of short hairpin RNA (shRNA) from plasmid vectors in BHK cells [56]. Similarly, when cell lines demonstrating ribozyme hairpin-mediated RNA cleavage activity were transfected with SIN particles, viral replication was significantly inhibited [57]. Furthermore, SIN-based expression of the transcription factor Broad-Complex (BR-C) antisense RNA in the silkmoth *Bombyx morii* reduced the endogenous BR-C expression and resulted in the failure of larval-pupal transition [58]. Likewise, SIN-mediated RNAi expression of the GATA factor revealed a link to anautogeny in *Aedes aegypti* mosquitoes [59].

In another approach, tissue-specific miRNA sequences were introduced into an alphavirus vector with the aim of altering tissue tropism for viral replication [60]. Introduction of six tandem targets for the neuron-specific miR124 between the nsP3 and nsP4 genes in replicative SFV4 vectors demonstrated attenuated spread into the CNS in BALB/c mice, which significantly prolonged the survival time of animals. Taken together, alphavirus-based RNAi along with the highly efficient RNA delivery and replication capacity, make alphaviruses potential candidates for RNAi-based gene therapy.

## 5. Conclusions

Alphavirus vectors based on naked RNA replicons, layered DNA/RNA vectors, and recombinant viral particles have demonstrated efficient gene delivery and recombinant protein expression in both cell lines and animal models. However, the shortcomings of the application of alphavirus vectors relates to the relatively expensive virus stock production and the highly transient nature of heterologous gene expression. In the context of virus production, packaging cell lines have been engineered [61,62], which has allowed alphavirus production from packaging cells, albeit with approximately 100-fold reduced titers. Despite the engineering of novel less cytotoxic alphavirus vectors, which provide prolonged expression, alphavirus vectors possess serious limitations in case of need of long-term therapeutic efficacy. On the other, for demands of short-term expression, therapeutic efficacy has been demonstrated in mouse tumor models after intratumoral vector administration. Interestingly, SIN vectors can also provide natural tumor targeting, which has enhanced therapeutic applications. In parallel, tumor targeting through lipid encapsulation of SFV RNA and particle formulations has enhanced specific tumor delivery. Furthermore, application of replication-proficient oncolytic alphavirus vectors has proven useful for improved delivery and prolonged therapeutic effect. The use of alphavirus vectors has proven highly successful for immunization with tumor antigens, which has provided both prophylactic and therapeutic efficacy in animal tumor models. Much effort has also been dedicated to the development of alphavirus vectors with reduced cytotoxicity and enhanced gene expression capacity to improve therapeutic efficacy and duration. Tumor vaccine development based on alphavirus RNA, plasmid DNA, and recombinant particles has further provided strong immune responses in animal models and protection against tumor challenges. So far, a limited number of alphavirus-based clinical trials have been conducted with liposome-encapsulated SFV particles and VEE vectors carrying carcinoembryonic antigen (CEA). The recent rapid development of RNAi and the discovery of the association of a large number of miRNAs with cancer should accelerate the application of alphavirus vectors in gene therapy.
